# Cognitive Impairment and Metabolite Profile Alterations in the Hippocampus and Cortex of Male and Female Mice Exposed to a Fat and Sugar-Rich Diet are Normalized by Diet Reversal

**DOI:** 10.14336/AD.2021.0720

**Published:** 2022-02-01

**Authors:** Alba M Garcia-Serrano, Adélaïde A Mohr, Juliette Philippe, Cecilia Skoug, Peter Spégel, João M.N Duarte

**Affiliations:** ^1^Department of Experimental Medical Science, Faculty of Medicine, Lund University, Lund, Sweden.; ^2^Wallenberg Centre for Molecular Medicine, Lund University, Lund, Sweden.; ^3^Institute of Physics, School of Basic Sciences, École Polytechnique Fédérale de Lausanne (EPFL), Lausanne, Switzerland.; ^4^Department of Chemistry, Centre for Analysis and Synthesis, Lund University, Lund Sweden

**Keywords:** brain metabolism, memory, anxiety, obesity, diabetes, sucrose, high-fat

## Abstract

Diabetes impacts on brain metabolism, structure, and function. Alterations in brain metabolism have been observed in obesity and diabetes models induced by exposure to diets rich in saturated fat and/or sugar and have been linked to memory impairment. However, it remains to be determined whether brain dysfunction induced by obesogenic diets results from permanent brain alterations. We tested the hypothesis that an obesogenic diet (high-fat and high-sucrose diet; HFHSD) causes reversible changes in hippocampus and cortex metabolism and alterations in behavior. Mice were exposed to HFHSD for 24 weeks or for 16 weeks followed by 8 weeks of diet normalization. Development of the metabolic syndrome, changes in behavior, and brain metabolite profiles by magnetic resonance spectroscopy (MRS) were assessed longitudinally. Control mice were fed an ingredient-matched low-fat and low-sugar diet. Mice fed the HFHSD developed obesity, glucose intolerance and insulin resistance, with a more severe phenotype in male than female mice. Relative to controls, both male and female HFHSD-fed mice showed increased anxiety-like behavior, impaired memory in object recognition tasks, but preserved working spatial memory as evaluated by spontaneous alternation in a Y-maze. Alterations in the metabolite profiles were observed both in the hippocampus and cortex but were more distinct in the hippocampus. HFHSD-induced metabolic changes included altered levels of lactate, glutamate, GABA, glutathione, taurine, *N*-acetylaspartate, total creatine and total choline. Notably, HFHSD-induced metabolic syndrome, anxiety, memory impairment, and brain metabolic alterations recovered upon diet normalization for 8 weeks. In conclusion, cortical and hippocampal derangements induced by long-term HFHSD consumption are reversible rather than being the result of permanent tissue damage.

The incidence of neurodegenerative diseases is increasing worldwide in its sporadic forms, which is likely due to increased longevity and unhealthy lifestyles [1]. In particular, obesity is associated closely to comorbidities such as cardiovascular disease, metabolic syndrome and type 2 diabetes (T2D) [2], disorders that impact the brain and have been linked to mild cognitive impairment, Alzheimer´s disease or vascular dementia [3,4]. Mechanisms of brain dysfunction linked to obesity and metabolic disorders are suggested to include glucose toxicity, vascular dysfunction, mitochondrial damage, oxidative stress, synaptic failure or neuroinflammation [5]. In rodent models, obesity and metabolic syndrome have been linked with alterations in brain function [6] and metabolism [5].

Magnetic resonance spectroscopy (MRS) enables non-invasive profiling of regional metabolic profiles [7]. Diabetes and associated comorbidities have been shown to cause variations in brain concentrations of *N*-acetylaspartate, creatine, choline, *myo*-inositol, glutamate and glutamine [7,8]. Alterations in metabolism have also been observed in multiple diabetes models [9-13], and in rodents that develop metabolic syndrome upon long-term exposure to obesogenic diets [14-16]. While the metabolic syndrome caused by long-term exposure to obesogenic diets is reversible [17], it remains to be determined whether associated alterations in brain metabolism are plastic, or the result from major structural injury to brain tissue. We aimed at testing the hypothesis that a long-term high-fat and high-sucrose diet (HFHSD) exposure causes reversible alterations of brain function (behavior) and metabolism.

**Figure 1.**
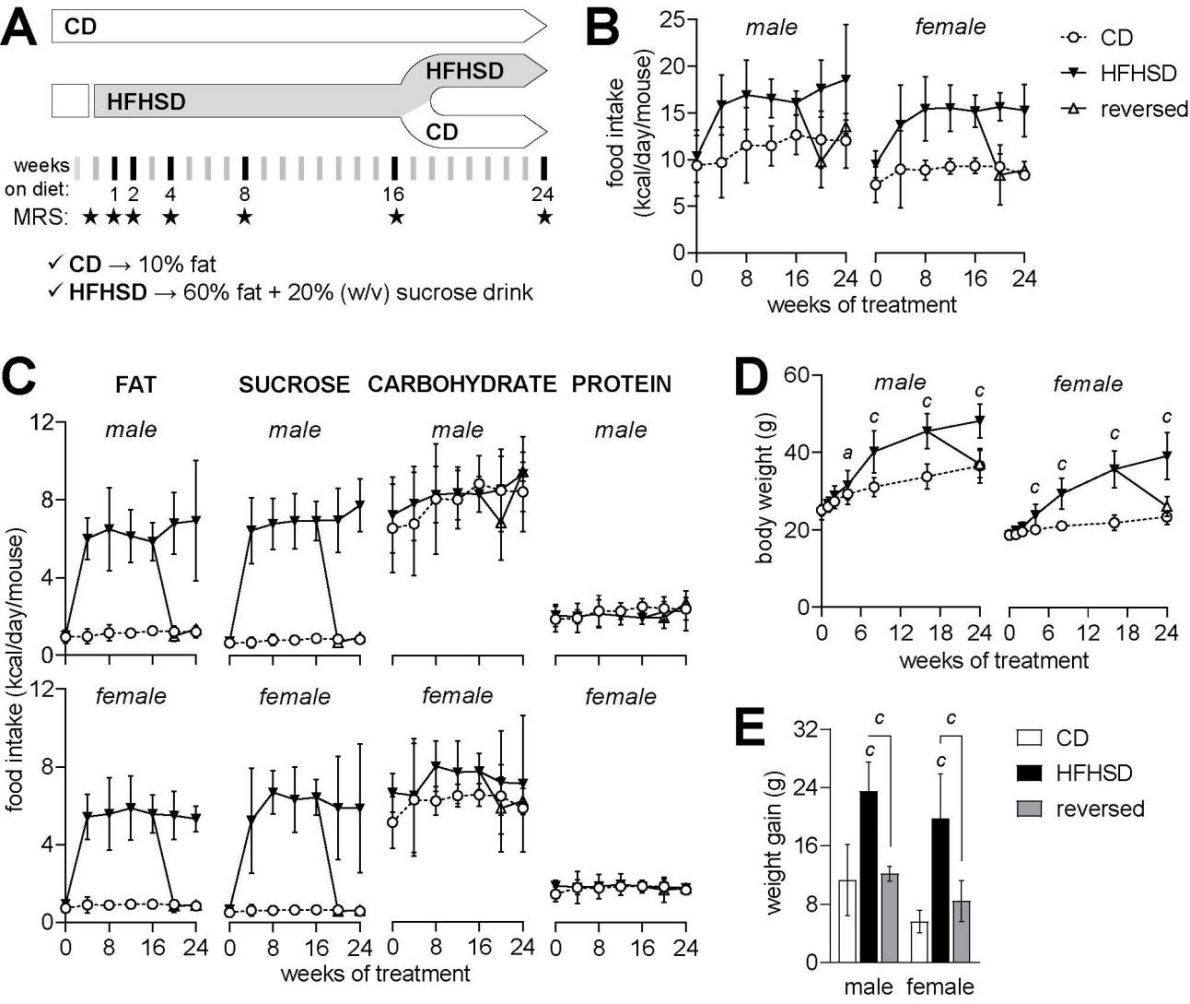
Study design and caloric intake in each month of the treatment. Mice were acclimatized under control diet (CD) at 8 weeks of age and exposed to HFHSD for 4 or 6 months starting at 10 weeks of age (A). A group of mice had the diet reversed to control after 4 months under HFD (reversed). MRS scans took place at baseline, and then at weeks 1, 2, 4, 8, 16 and 24 of the treatment (stars in timeline of panel A). Average caloric intake from increased during HFHSD-feeding (B) due to increased fat and sucrose intake (C). Relative to CD, HFHSD feeding resulted in increased body weight (D) and an over 2-fold larger weight gain from baseline to 6 months of treatment (E), which is fully reversed by diet normalization. Data is mean±SD. Letters over data-points indicate significant differences relative to CD or as indicated (*^a^* P<0.05, *^b^* P<0.01, *^c^* P<0.001) based on Fisher’s LSD *post hoc* comparison for significant effects of diet or interaction between diet and time, as assessed by ANOVA. Food intake is the cage average (n=3-9).

## MATERIALS AND METHODS

### Animals

Experiments were performed according to EU Directive 2010/63/EU, approved by the Malmö/Lund Committee for Animal Experiment Ethics (#994/2018), and are reported following the ARRIVE guidelines (Animal Research: Reporting In Vivo Experiments, NC3Rs initiative, UK). Sample size was estimated from previous MRS studies [15,18].

Male and female C57BL/6J mice (8-weeks old) were purchased from Taconic Biosciences (Köln, Germany), and housed in groups of 3-5 animals on a 12h light-dark cycle with lights on at 07:00, room temperature of 21-23 °C and humidity at 55-60%. Mice were habituated to the facility for 1 week upon arrival.

Mice were randomly assigned to 3 experimental groups, receiving either a control diet (CD, 10%-fat diet, n=12 males + 15 females), a high-fat and high-sucrose diet (HFHSD, n=13 males + 13 females) consisting of 60%-fat diet feeding plus access to a 20%(w/v) sucrose in drinking water from 10 weeks of age and during 6 months, or a reversed diet (reversed, n=9 males + 10 females) that consisted of HFHSD feeding for 4 months followed by CD feeding for 2 months (Fig. 1A). Mice receiving HFHSD also had access to sugar-free water. Food and water were provided *ad libitum*. Brain tissue samples were collected after experiments *in vivo* at 24 weeks (see below).

This study used open-source diets from Research Diets (New Brunswick, NJ-USA): a lard-based diet with 60% kcal of fat (D12492) and a control diet containing 10% kcal of fat (D12450J), with total energy of 5.21 and 3.82 kcal/g, respectively. Based on the relative amounts of fat sources (lard/soybean oil), the saturated, monounsaturated and polyunsaturated fatty acid distribution was estimated to be 27%, 36% and 38% in CD, and 38%, 48% and 14% in HFHSD, respectively. Both diets contain 20% kcal from protein and 7% kcal from sucrose, and the remaining calories are from carbohydrates (maltodextrin and starch). Such 60%-fat diet was more effective at eliciting brain metabolic alterations (relative to the control diet) than a composition-matched 45%-fat diet [15].

### Magnetic Resonance Spectroscopy (MRS)

MRS was performed at baseline and at 1, 2, 4, 8, 16 and 24 weeks after initiation of the diet. For 6 mice in each group, MRS was only conducted at 16 and 24 weeks. Experiments were performed on a 9.4T Bruker BioSpec AV III (Bruker, Ettlingen, Germany) with an effective bore size of 86 mm and gradient strength of 670mT/m, using ParaVision 6.0.1 (RRID:SCR_001964), and equipped with a ^1^H quadrature transmit/receive cryoprobe. Anesthesia was induced with 3% isoflurane (Vetflurane, Virbac, Carros, France) in a 1:1 (v/v) O_2_:N_2_O gas mixture. Then mice were positioned onto a MRI-compatible bed with teeth and ear bars for stereotaxic fixation. Anesthesia was delivered through a home-build mask at variable rate of 1-2% isoflurane for maintaining respiration at 60-90 breaths per minute. Body temperature was kept at 36-37ºC by means of warm water circulation. Breathing rate and body temperature were recorded with the SA Instruments (Stony Brook, NY, USA) monitoring system.

T_2_-weighted images were acquired for anatomical reference using the Rapid Imaging with Refocused Echoes (RARE) sequence with repetition time TR=3.5 s, echo time TE=33 ms, echo train length of 8, 32 slices, thickness of 0.5 mm, 320x320 voxels, and FOV=14×14 mm^2^. After MAPSHIM (Bruker) and iterative linear shimming, proton MRS was acquired using STimulated Echo Acquisition Mode (STEAM) with TR=4 s, TE=3 ms, mixing time TM=20 ms, and a spectral width of 4401.41 Hz. Volumes of interest (VOI) for MRS were placed in dorsal hippocampus (1.8 mm x 1.2 mm x 1.5 mm) and cortex (4 mm x 0.8 mm x 1.5 mm).

Water-suppressed spectra were acquired in 20 and 12 blocks of 16 scans in the hippocampus and cortex, respectively. Unsuppressed water spectra were acquired from the same VOIs in one block of 16 averages. After block alignment and summation, metabolite concentrations were determined with LCModel v.6.3-1A (Stephen Provencher Inc., Oakville, Ontario-Canada; RRID:SCR_014455), including a macromolecule spectrum in the database and using the unsuppressed water signal as internal reference [19]. The following metabolites were included in the LCModel analysis: alanine, ascorbate, aspartate, β-hydroxybutyrate, creatine, γ-aminobutyrate (GABA), glutamine, glutamate, glutathione, glycine, glycerophosphorylcholine (GPC), glucose, lactate, *myo*-inositol, *N*-acetylaspartate, *N*-acetylaspartylglutamate (NAAG), phosphoryl-ethanolamine (PE), phosphorylcholine (PCho), phosphocreatine, *scyllo*-inositol, and taurine. After LCModel analysis, metabolites with Cramér-Rao lower bound (CRLB) larger than 30% were disregarded as they did not fulfil reliability criteria. Namely, β-hydroxybutyrate and *scyllo*-inositol were excluded, and phosphorylcholine and glycerophosphorylcholine were analyzed as total choline (PCho+GPC).

Spectra showing extra-cerebral lipid contamination were excluded from the analysis (36 out 828 MRS scans). At the end of the study, one mouse was excluded due to simultaneous increase of glutamine levels and decreased osmolytes in both brain areas and at all MRS time points, suggesting occurrence of a congenital portosystemic shunt [20].

**Figure 2.**
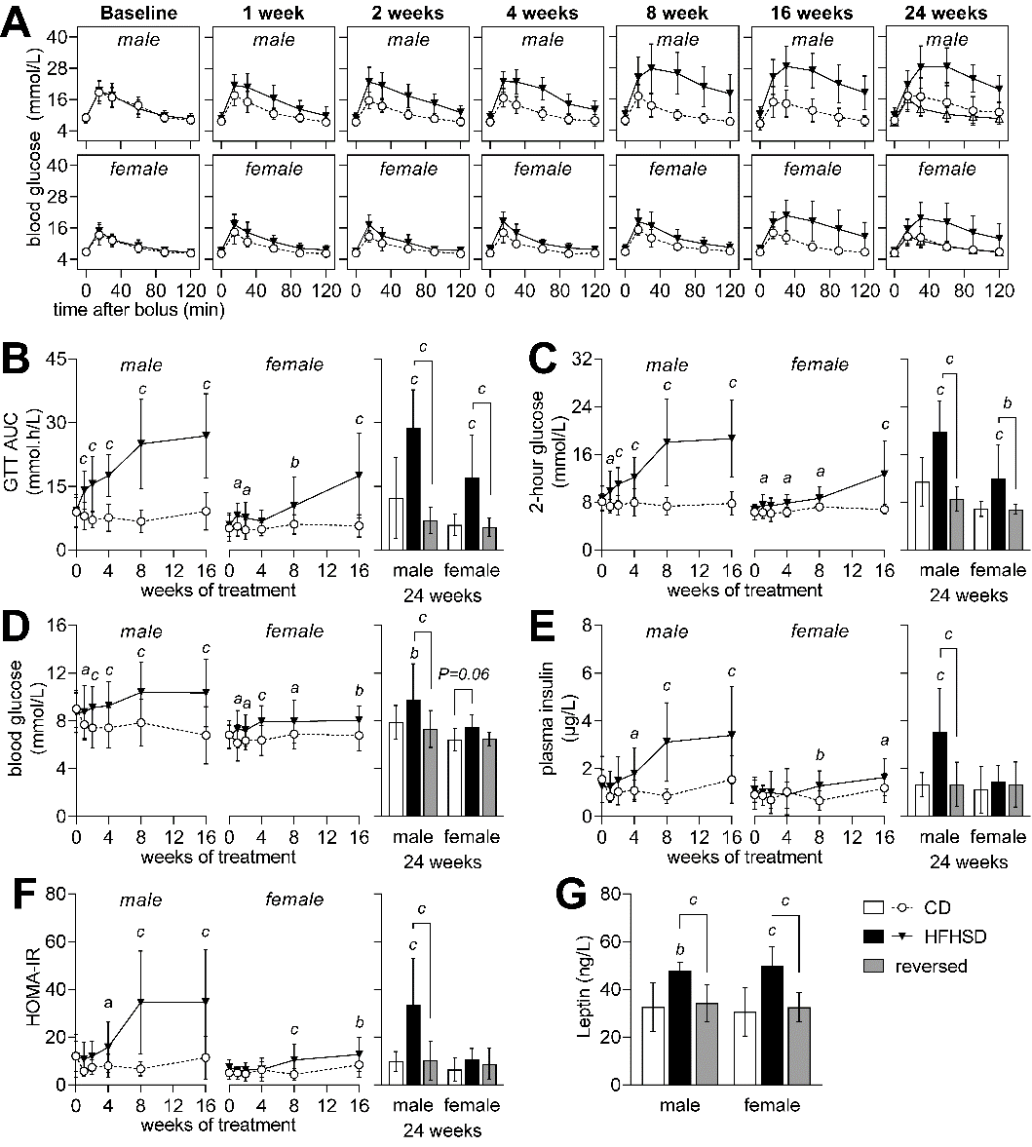
Glycemic regulation in mice from control (CD, open circles), HFHSD (filed triangles) and reversed (open triangles) groups at baseline and after 1, 2, 4, 8, 16 and 24 weeks of the treatment. Glucose clearance (A) in a glucose tolerance test (GTT) was reduced by HFHSD feeding, as evidenced by both increased area under the curve (AUC) of the GTT (B) and increased glycemia 2 hours after the glucose bolus (C). Blood glucose (D), plasma insulin (E) and HOMA-IR (F) after a 6-hour fasting period indicate insulin resistance in HFHSD-fed male mice. Compared to controls, plasma leptin was increased in HFHSD-fed mice and normalized by diet reversal (G). Data is mean±SD. Letters over data-points indicate significant differences relative to CD or as indicated (*^a^* P<0.05, *^b^* P<0.01, *^c^* P<0.001) based on Fisher’s LSD *post hoc* comparison following presence of significant effects of diet or interaction between diet and time in ANOVA tests.

### Behavior

Mice were allowed to acclimatize to the testing room for 1 hour. Tests were performed from 9:00 to 18:00, with light adjusted to an illuminance of 15 lx in each apparatus.

Spontaneous alternations were observed in a Y-maze as surrogate of working memory performance [21], at baseline and after 1, 2, 4, 8, 16 and 24 weeks of treatment. The Y-maze arms were 30 cm x 15 cm x 5 cm (length x height x width) and converged to the center at an 120° angle. Mice were placed in one arm and allowed to freely explore the maze for 8 minutes. Complete spontaneous alternations were defined as successive entries into the three arms and expressed relative to the total possible alternations in the test. The total number of entries was used to access locomotor activity and exploratory behavior.

Object recognition tasks were adapted from de Paula *et al*. [22], in which memory is tested based on the spontaneous tendency of rodents familiarized with two objects to explore a novel object (novel object recognition, NOR) or an object relocated in space (novel location recognition, NLR). Exploration was recorded by an infrared camera in a cubic arena with a side length of 50 cm. Mice were first habituated to the empty arena for 8 minutes. Arena exploration was analyzed for total walk distance, number of crossings between arena quadrants and immobility time, as well as exploration of the arena center at 6 cm from the walls. Thereafter, NLR was assessed by placing the mice in the arena with two identical objects and allowed to explore them for 5 minutes (familiarization phase). Mice were then removed from the arena for 1 hour (retention phase) and reintroduced for 5 minutes but with one object relocated to a different quadrant in the arena (recognition phase). For NOR, two new identical objects were used in the familiarization phase, and one of them was replaced by a novel object during the recognition phase. Time exploring each object was measured.

### Glucose tolerance test (GTT) and hormone analyses

A GTT was performed before (baseline) and after 1, 2, 4, 8, 16 and 24 weeks on the diets. Food was removed for 6 hours starting at 08:00. Thereafter, a blood sample was collected from the vena saphena to determine plasma hormone levels. Glycemia was measured from tail tip blood with the Breeze glucometer (Bayer, Zürich, Switzerland). Then mice were given 2 g/kg glucose i.p. from a 30%(w/v) solution in saline, followed by determination of glucose levels at 15, 30, 60, 90, and 120 minutes. Commercially available ELISA kits were used to determine plasma concentrations of insulin (#10-1247-10, Mercodia, Uppsala, Sweden; RRID:AB_2889906) and leptin (#ab100718, Abcam, Cambridge, United Kingdom; RRID:AB_2889903).

### Immunofluorescence confocal microscopy

Mice under isoflurane anesthesia were sacrificed by cardiac perfusion with cold PBS and then cold phosphate-buffered formaldehyde (Histolab, Askim, Sweden), and brains were cryosectioned into 20 µm slices [21]. Immunolabeling was carried out as detailed previously [23] with the primary antibodies: rabbit anti-allograft inflammatory factor 1 (Iba1, dilution 1:200; #019-19741, Fujifilm Wako, Japan; RRID:AB_839504), rat anti-CD11b (dilution 1:500; #MCA711, Bio-Rad, Sundbyberg, Sweden; RRID:AB_321292), anti-glial fibrillary acidic protein (GFAP) pre-tagged with AF488 (dilution 1:500; #53-9892-82, ThermoFisher Scientific, Göteborg, Sweden; RRID:AB_10598515), rabbit anti-glutamine synthetase (dilution 1:250; #ab176562, Abcam, Cambridge, UK; RRID:AB_2868472), anti-NeuN pre-tagged with AF488 (dilution 1:100; Cat# ab190195, Abcam; RRID:AB_2716282) and rabbit anti-doublecortin (dilution 1:500; #AB2253, Millipore-Merck, Darmstadt, Germany; RRID:AB_1586992). Secondary antibodies (dilution 1:500) were from ThermoFisher: AF568-conjugated goat anti-Rabbit IgG (#A-21069; RRID: AB_141416), AF568-conjugated goat anti-rat IgG (#A-21247; RRID:AB_141778) and AF488-conjugated goat anti-guinea-pig IgG (#A-11073; RRID:AB_2534117). After mounting, slices were examined under a Nikon A1RHD confocal microscope (Nikon Instruments, Tokyo, Japan). Images were acquired with NIS-element v5.20.01 (Laboratory Imaging, Nikon), and analyzed in ImageJ (NIH, Bethesda, MD, USA).

### Immunoblotting

Western blot was carried out as detailed previously [15] with antibodies against Iba1 (#016-200001, Fujifilm Wako; RRID: AB_2892192), GFAP (#ab68428, Abcam; RRID: AB_1209224) and β-actin (#ab6276, Abcam; RRID:AB_2223210).

### Real-time polymerase chain reaction (RT-PCR)

RNA was isolated from the hippocampus with Trizol (#15596026, Invitrogen, USA), and then 1 µg of total RNA was reverse transcribed with random hexamer primers using the qScript cDNA SuperMix (#95048, Quantabio, England), according to the manufacturers’ instructions. The resulting cDNA was used for quantitative RT-PCR as detailed by Jujic *et al.* [24] using PerfeCTa SYBRgreen SuperMix (#95054, Quantabio, England) and primers for IL-10 (forward: ATGGTGTCCTTTCAATTGCTC, reverse: AGGATCT CCCTGGTTTCTCTT), IL-6 (forward: TCTGAAGGAC TCTGGCTTTG, reverse: GATGGATGCTACCAAAC TGGA), IL-1β (forward: GAAGAGCCCATCCTCTG TGA, reverse: TTCATCTCGGAGCCTGTAGTG), NF-κβ (forward: CGGAGGACGGAGACTCGTT, reverse: CCATGGTCAGCGGCTTCT), TNF-α (forward: TTGA CCTCAGCGCTGAGTTG, reverse: CCTGTAGCCCAC GTCGTAGC), or the 60S ribosomal protein L14 (forward: GGCTTTAGTGGATGGACCCT, reverse: ATTGATATCCGCCTTCTCCC). Gene expression was normalized to L14 expression analyzed with the comparative cycle threshold (CT) method (ΔΔCT).

**Figure 3.**
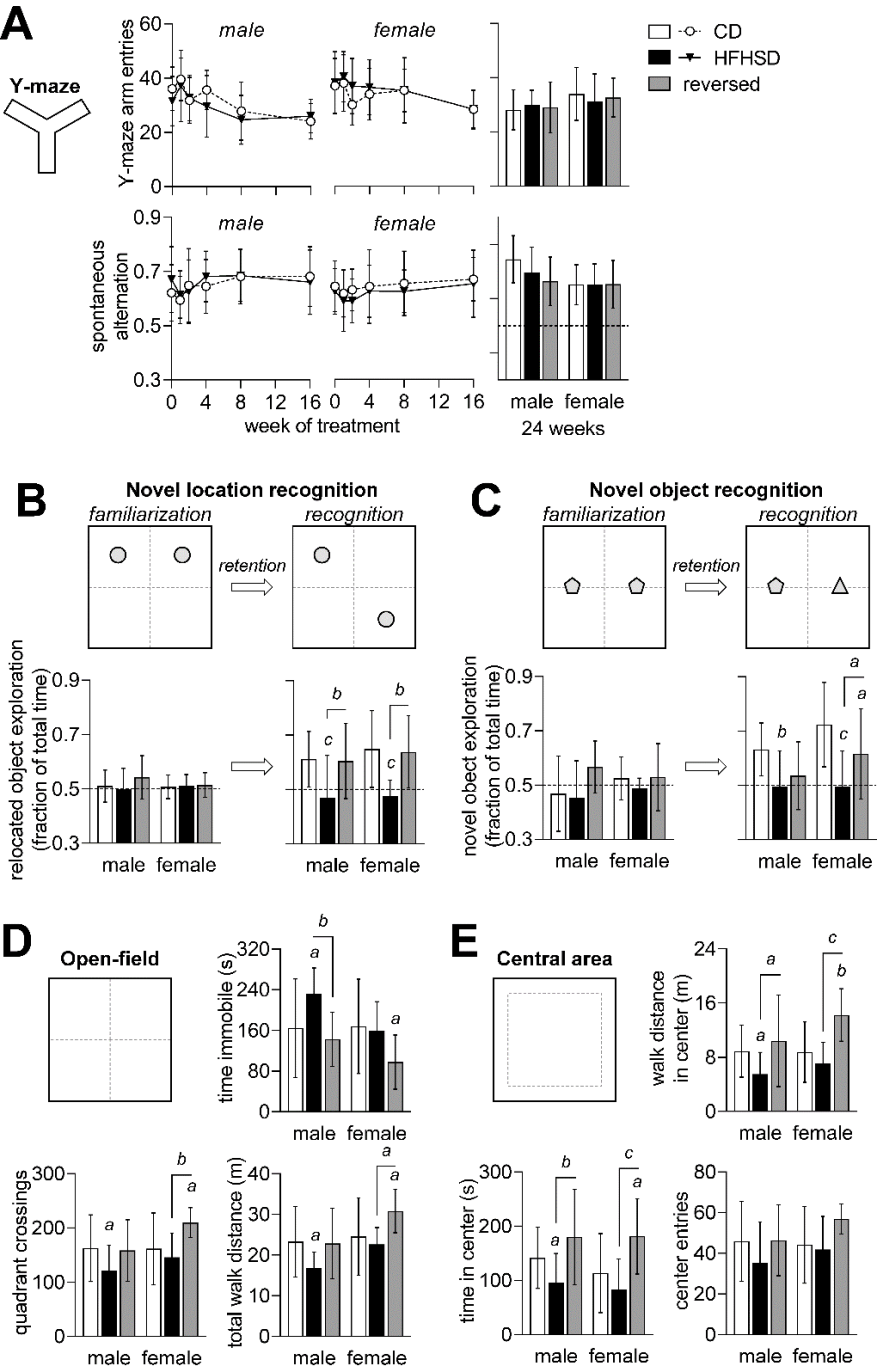
Memory impairment induced by HFHSD exposure. Number of arm entries in the Y-maze and spontaneous alternation (A) were not modified during exposure to HFHSD. After familiarization with 2 objects, both novel location recognition (B) and novel object recognition (C) tasks show that mice exposed to HFHSD for 24 weeks did not display increased exploration of novel location or object. When diet was reversed, mice recovered the ability to recognize novelty in the object displacement task (D) but not in the object replacement task (E). Dashed lines in graphs represent chance (50%). Data is mean±SD. Letters over data-points indicate significant differences relative to CD or as indicated (*^a^* P<0.05, *^b^* P<0.01, *^c^* P<0.001) based on Fisher’s LSD *post hoc* comparison following presence of significant effects of diet or interaction between diet and time in ANOVA tests.

### Statistics

Data were analyzed by ANOVA followed by independent comparisons with the Fisher’s least significant difference (LSD) test in Prism 9.0.2 (GraphPad, San Diego, CA-US; RRID:SCR_002798). Principal component analysis (PCA) was conducted in R 3.6.2 using prcomp (RRID:SCR_001905). Results are presented as mean±SD unless otherwise stated.

## RESULTS

### Food intake and metabolic phenotype

Mice on HFHSD had a higher caloric intake as compared to mice on the control diet (Fig. 1B), which was due increased consumption of fat and sucrose, rather than carbohydrates or protein (Fig. 1C). Consequently, mice fed HFHSD became overweight, but then returned to normal levels after a transition to the control diet (Fig. 1D). Weight gain from baseline to the end of the study was higher in HFHSD-fed mice, but not in the group of mice for which the diet was reversed, relative to controls (diet P<0.001; gender P<0.001; interaction P=0.657; Fig. 1E).

In line with previous studies in mice fed obesogenic diets [17], the HFHSD caused sex-dependent imbalances in glucose homeostasis, which were fully recovered after diet reversal (Fig. 2). Interestingly, at the end of the 24 weeks of treatment, HFHSD-associated glucose intolerance was more severe in male than female mice (Fig. 2B-C), and insulin resistance was negligible in females (Fig. 2F). Glucose clearance during the GTT (Fig. 2A), as defined by both the glucose area-under-the-curve (at 24 weeks: diet P<0.001; gender P<0.001; interaction P=0.056; Fig. 2B) and glucose levels at 2 hours post-bolus (at 24 weeks: diet P<0.001; gender P<0.001; interaction P=0.025; Fig. 2C), was impaired by HFHSD feeding.

Mice fed HFHSD showed slightly higher fasting glucose (diet P<0.001; gender P<0.001; interaction P=0.292; Fig. 2D) and plasma insulin levels (diet P<0.001; gender P=0.011; interaction P=0.007; Fig. 2E) than controls. This translated into an increased HOMA-IR (Homeostatic Model Assessment for Insulin Resistance), indicative of insulin resistance (diet P<0.001; gender P=0.001; interaction P=0.003; Fig. 2F). Plasma leptin was increased in HFHSD-fed mice but not in the diet reversal group, relative to controls (diet P<0.001; gender P=0.815; interaction P=0.801; Fig.1G). Altogether, these results suggest that obesity induced by HFHSD feeding causes glucose intolerance and insulin resistance, which were normalized by diet reversal.

**Figure 4.**
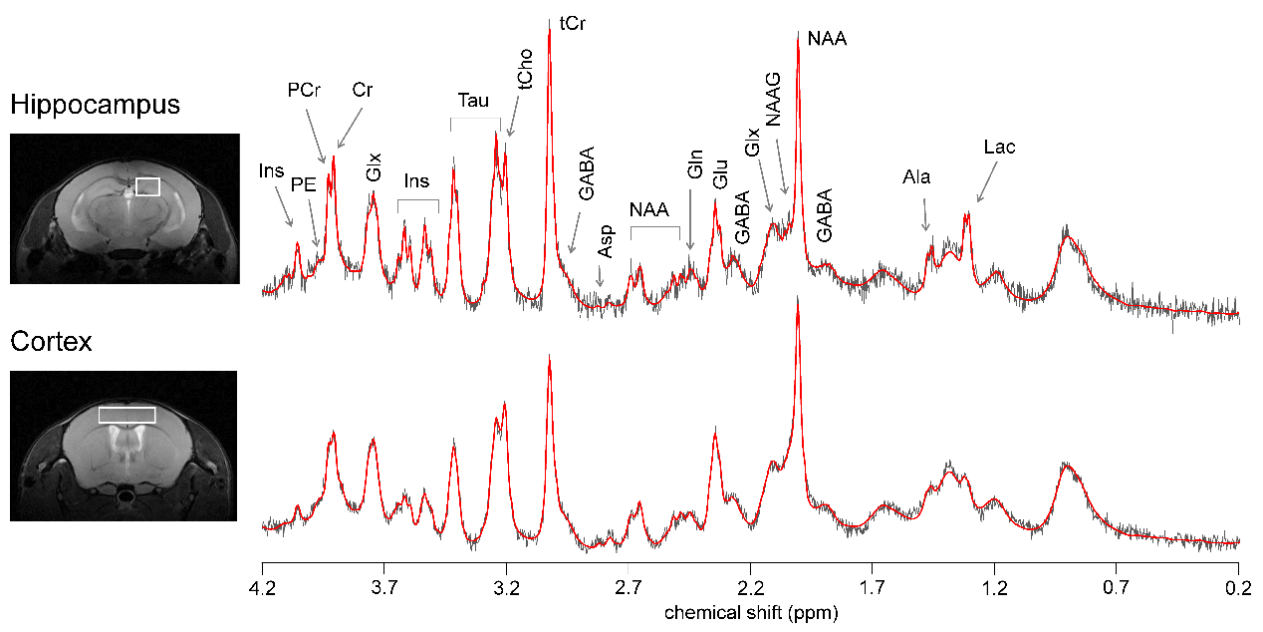
Representative location of the VOIs used for MRS in dorsal hippocampus and cortex, and respective spectra acquired with STEAM at 9.4 T (gray line) and LCModel fitting result (red line). Spectra were acquired with 320 and 192 averages in the hippocampus and cortex, respectively. From right to left: Ala, alanine; Lac, lactate; GABA, γ-aminobutyrate; NAA, *N*-acetylaspartate; NAAG, *N*-acetylaspartylglutamate; Glx, glutamate (Glu) + glutamine (Gln); Asp, aspartate; tCr, total creatine = creatine (Cr) + phosphocreatine (PCr); tCho, total choline = phosphorylcholine + glycerophosphorylcholine; Tau, taurine; Ins, *myo*-inositol; PE, phosphorylethanolamine.

**Figure 5.**
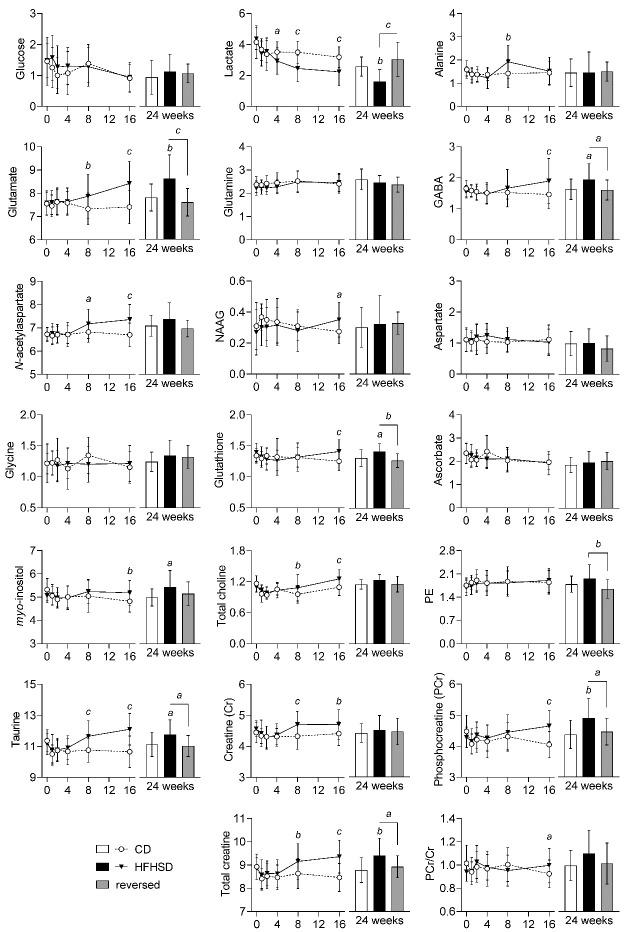
Alterations in metabolite concentrations (in µmol/g) triggered by HFHSD feeding in the hippocampus. Data is mean ± SD. Letters over data-points indicate significant differences relative to CD or as indicated (*^a^* P<0.05, *^b^* P<0.01, *^c^* P<0.001) based on Fisher’s LSD *post hoc* comparison following presence of significant effects of diet or interaction between diet and time in ANOVA tests.

**Figure 6.**
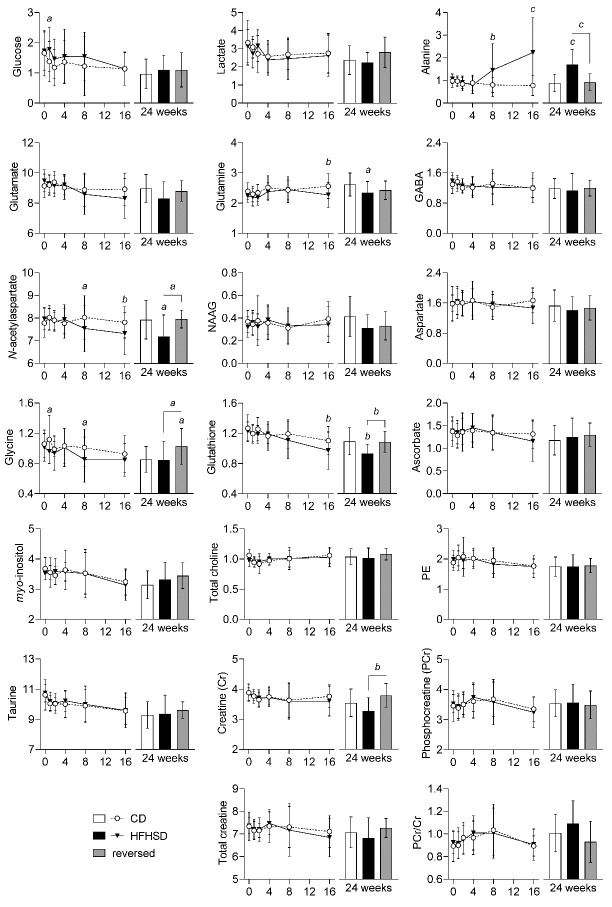
Alterations in metabolite concentrations (in µmol/g) triggered by HFHSD feeding in the cortex. Data is mean ± SD. Letters over data-points indicate significant differences relative to CD or as indicated (*^a^* P<0.05, *^b^* P<0.01, *^c^* P<0.001) based on Fisher’s LSD *post hoc* comparison following presence of significant effects of diet or interaction between diet and time in ANOVA tests.

### HFHSD-induced behavior alterations

Having established a reversible diet-dependent effect on glycemic control, we then investigated whether metabolic syndrome was associated with behavioral alterations. Spatial working memory was evaluated by measuring Y-maze spontaneous alternation during the course of phenotype development (Fig.3A). Diet had no effect on the spontaneous alternation (at 24 weeks: diet P=0.353; gender P=0.034; interaction P=0.334), or exploratory behavior depicted by number of arm entries (at 24 weeks: diet P=0.638; gender P=0.116; interaction P=0.988).

Next, we examined novelty recognition at 24 weeks after introduction of the diets. After familiarization with 2 objects, mice normally tend to spend more time exploring a novel object, or an object that has been displaced in the arena. This behavior was impaired in HFHSD-fed mice when testing either novel location recognition (diet P<0.001; gender P=0.341; interaction P=0.867; figure 3C) or novel object recognition (diet P<0.001; gender P=0.059; interaction P=0.308; Fig. 3D). relative to HFHSD, the reversed diet group fully recovered memory performance to control levels in the novel location recognition task (Fig. 3C), and also improved performance in the novel object recognition task (Fig. 3D).

We examined open-field exploration for 8 minutes (habituation prior to object recognition tasks). HFHSD exposure reduced total distance walked in the arena (diet P=0.006; gender P=0.004; interaction P=0.224; Fig. 3E). Moreover, when compared to controls, male mice fed HFHSD showed reduced number of crossings across quadrants of the arena (diet P=0.008; gender P=0.047; interaction P=0.234), and increased immobility time (diet P=0.006; gender P=0.030; interaction P=0.121). Altogether, these results suggest reduced novel environment exploration upon HFHSD exposure, which is normalized after diet reversal.

Typically, rodents spend a greater amount of time exploring the periphery of the arena, next to the walls, rather than the unprotected center area [25]. HFHSD-fed mice spent less time (diet P<0.001; gender P=0.375; interaction P=0.716; Fig. 3F) and walked a shorter distance in the center of the arena (diet P<0.001; gender P=0.071; interaction P=0.256), despite similar number of center entries (diet P=0.055; gender P=0.214; interaction P=0.435). Reduced exploration of center *versus* periphery suggests increased anxiety-like behavior in HFHSD-fed mice [25], which recovered upon diet normalization.

### Metabolite profiles

Changes in behavior were similar in male and female mice (Fig. 3). Moreover, we have previously demonstrated that metabolite profiles measured by MRS in the hippocampus and cortex are similar in male and female mice [19]. Thus, in order to increase the statistical power, metabolite profiles were analyzed for male and female mice grouped together. Figure 4 depicts the VOI placement in hippocampus and cortex, and respective spectra.

**Figure 7.**
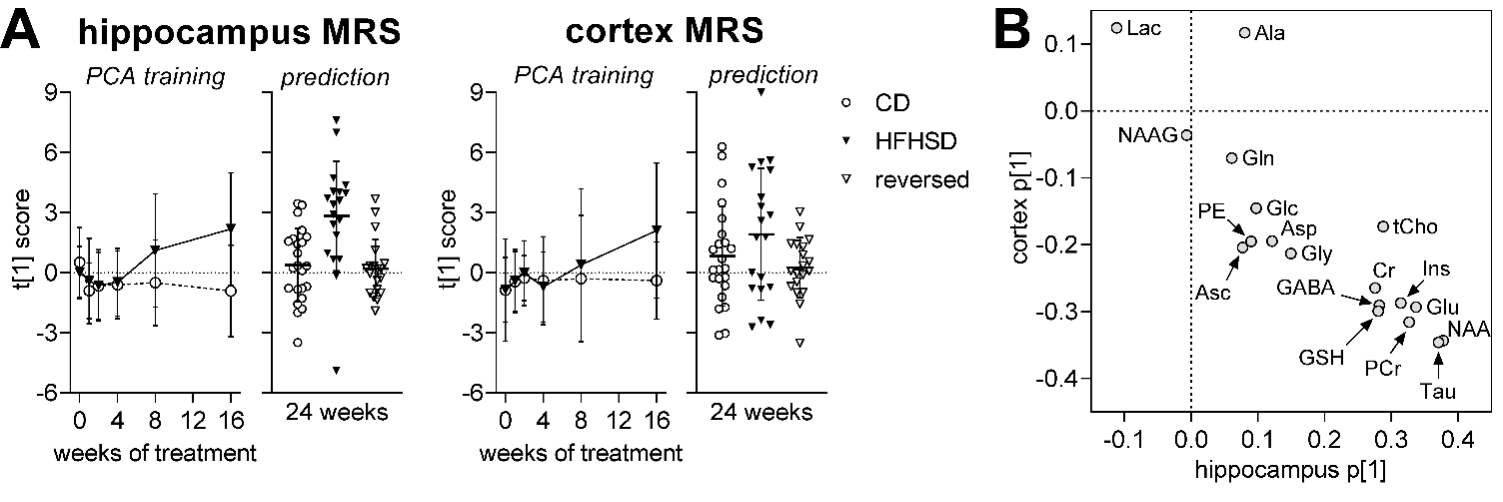
Alterations in the overall metabolite profile in hippocampus and cortex examined using principal component analysis (PCA). The PCA was calculated for the first 16 weeks (A). Trajectories are based on the scores along the first principal component (PC1), describing time-dependent alterations in the metabolite profile. Scores were then estimated from metabolite levels measured at 24 weeks of examination (prediction), revealing a normalization of the metabolite profile after diet reversal. Variation explained by the first principal component (PC1) was 29% in hippocampus and 36% in cortex. (B) Weights for each metabolite along PC1 for hippocampus and cortex. Data is individual data-points or mean ± SD.

**Figure 8.**
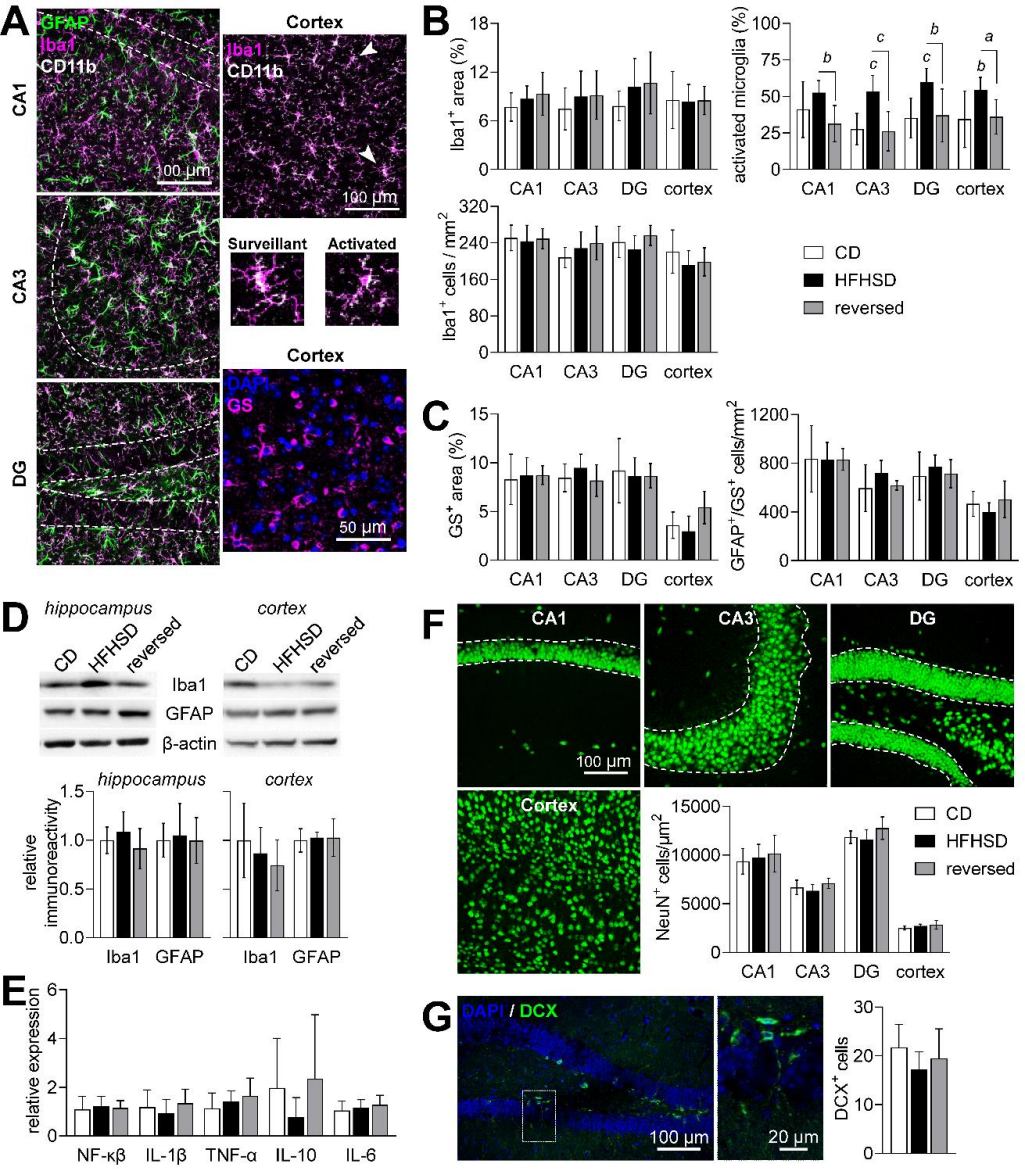
Neuroinflammation and neurodegeneration analysis in the hippocampus and cortex. (A) Confocal micrographs depicting Iba1-, CD11b- and GFAP-immunolabeled cells in the cornus amonis CA1/CA3 and dentate gyrus (DG) of the hippocampus or in the cortex, and cortical immunolabeling of glutamine synthetase (GS). Typical surveillant and activated microglia (Iba1^+^ and CD11b^+^) phenotypes are indicated by the arrowheads, and expanded below the cortex micrograph. (B) HFHSD had no impact on the Iba1^+^ area or number of microglia cells, but increased the fraction of activated microglia, which was normalized by diet reversal. (C) Astrocytes were considered all GS^+^ and/or GFAP^+^ cells. HFHSD had no impact on the area occupied by GS^+^ cells or number of astrocytes. (D) Total Iba1 and GFAP levels in the hippocampus or cortex were similar across the experimental groups. (E) Expression of NF-κβ and cytokines in the hippocampus, relative to the 60S ribosomal protein L14. (F) NeuN immunolabeling of neuronal somata was used to estimate the number of mature neurons in the cortex and within the granule cell layer of CA1, CA3 and DG. (G) Doublecortin (DCX)-immunolabeling was used to count immature neurons. DCX^+^ cells are estimated per DG within a stained brain slice. Dashed lines in micrographs define the granule cell layer in CA1, CA3 and DG. Data is plotted as mean±SD of n=6-8 (half of either gender). Letters over data-points indicate significant differences relative to CD or as indicated (*^a^* P<0.05, *^b^* P<0.01, *^c^* P<0.001) based on Fisher’s LSD *post hoc* comparison following presence of significant effects of diet or interaction between diet and time in ANOVA tests.

HFHSD feeding altered levels of multiple metabolites in both hippocampus and cortex (Supplementary Table 1). In the hippocampus (Fig. 5), the earliest metabolic alteration triggered by HFHSD was a decrease in levels of lactate (P<0.05 *vs*. control at 4 weeks of diet) that persisted until the end of the treatment. Levels of glutamate (P<0.01), *N*-acetylaspartate (NAA; P<0.05), taurine (P<0.001) and creatine (P<0.001), increased at 8 weeks, relative the controls, and remained high throughout the intervention. At 16 weeks, concentrations of GABA (P<0.001), *myo*-inositol (P<0.01), phosphocreatine (P<0.001) and glutathione (P<0.001) increased in the HFHSD-fed mice, as compared to controls. HFHSD feeding also caused transient changes in levels of alanine at 8 weeks (P<0.01) and of *N*-acetylaspartylglutamate (NAAG) at 16 weeks (P<0.05) of treatment. All these metabolic alterations in the hippocampus were lost 8 weeks after diet reversal (supplementary Table 2; Fig. 5).

In general, the metabolite profile in the cortex (Fig. 6) was less impacted by HFHSD-feeding as compared to the hippocampus, and changes were generally in opposite direction. While cortical lactate levels remained unaltered, alanine concentrations were increased from 8 weeks of treatment in HFHSD-fed mice compared to controls (P<0.01). When compared to controls, we further observed a HFHSD-induced decrease of cortical *N*-acetylaspartate (P<0.05) from 8 weeks onwards and of glutamine (P<0.01) and glutathione (P<0.01) from 16 weeks onwards. Cortical glycine concentration showed a transient increase at 1 and 8 weeks of HFHSD feeding (P<0.05 *vs*. controls). While glycine levels were similar in HFHSD-fed mice and controls at 24 weeks of treatment, they were increased by diet reversal (P<0.05 *vs*. control and *vs*. HFHSD).

Brain glucose levels depend on glycemia and rates of brain glucose uptake and consumption [26]. In line with increased glycemia, we observed a tendency of higher brain glucose levels in mice fed HFHSD, as compared to mice fed the control diet (Supplementary Table 1; Fig. 5-Fig. 6).

We used principal component analysis (PCA) to provide a global depiction of brain metabolism. This analysis revealed a brain metabolic shift (relative to controls) in the hippocampus starting at 8 weeks of HFHSD feeding (Fig. 7A). The PCA conducted on MRS data collected during the first 16 weeks of treatment was then used to calculate the PCA scores from metabolite profiles acquired at 24 weeks. This analysis revealed a normalization of the hippocampal metabolite profile after diet reversal (Fig. 7A). Results were less distinct for the cortex, in line with the less pronounced metabolic alterations observed in this region during the first 16 weeks of the dietary intervention (Fig. 7A). Taurine and NAA were depicted by the PCA as the most influential metabolites in the effect of HFHSD on the brain metabolite profiles (Fig. 7B).

### Analysis of neuroinflammation and neurodegeneration in HFHSD

Neuroinflammation has been reported to occur upon exposure to obesogenic diets [6]. We thus explored whether neuroinflammation and gliosis are patent after 24 weeks of HFHSD exposure using fluorescence microscopy for Iba1, CD11b, GFAP and glutamine synthetase (Fig. 8A). HFHSD had no impact on area occupied by microglia or on the number of microglia cells (cells positive to Iba1 and CD11b) in either cortex or hippocampus (Fig. 8B). In turn, there was a significant HFHSD-induced increase in the fraction of activated microglia (diet P<0.001; brain region P=0.221; interaction P=0.737), as morphologically defined by Fernández-Arjona *et al*. [27], which was normalized by diet reversal. HFHSD had no effect on the number of astrocytes in the cortex or hippocampus (Fig. 8C), as observed by immunolabeling of GFAP and glutamine synthetase (astrocyte specific enzyme). Note that glutamine synthetase was used as astrocytic marker because cortical astrocytes only displayed GFAP staining at the cortical surface and around some blood vessels. Area occupied by glutamine synthase in hippocampus and cortex (Fig. 8C) or by GFAP in hippocampus (not shown) was also similar across the regions analyzed. Immunoblotting against Iba1 and GFAP in protein extracts from the cortex and hippocampus was similar across the 3 groups, corroborating the absence of changes in total microglia and astrocyte area (Fig. 8D). Furthermore, we have not observed alterations in expression levels of the inflammation regulator NF-κβ or cytokines in the hippocampus (Fig. 8E).

NeuN immunolabeling revealed that HFHSD had no effect on the number of mature neurons in the cortex or the granule cell layer of the hippocampus (Fig. 8F). Moreover, no HFHSD-induced changes were observed in neuron counts within the molecular layer or hilus of the hippocampus (not shown). Furthermore, the number of doublecortin-positive cells (immature neurons) in the dentate gyrus of the hippocampus showed no variation across the experimental groups (Fig. 8G).

Altogether, these results suggest phenotypic microglia alterations upon long-term HFHSD feeding, in the absence of a pro-inflammatory phenotype, astrogliosis or neuronal loss.

## DISCUSSION

Obesogenic diets impact brain function [5]. However, it is hitherto unknown whether brain alterations are chronic or reversible by the introduction of a healthier diet. Hence, in the present study we tested the reversibility of HFHSD-elicited changes in brain metabolism and behavior. We found that metabolite profile alterations in the hippocampus and cortex develop gradually and are observable by MRS within a relatively short period of time, *i.e.* within 4 weeks of HFHSD feeding. Most importantly, brain metabolism and behavior largely normalized upon diet reversal, suggesting cellular adaptations to metabolic syndrome rather than major brain injury in HFHSD-fed mice. Moreover, despite of a more severe metabolic syndrome in HFHSD-fed male mice as compared to female mice, behavioral changes were similar, and both genders exhibited recovery potential upon diet reversal.

### Metabolic syndrome and brain function

The severity of the metabolic syndrome and brain dysfunction in mice subjected to obesogenic diets is known to depend on the amount and types of fat and sugar in the diet [5,6]. We implemented a model of hyper-caloric feeding in which additional calories originate mainly from saturated fat in a lard-based diet and from a sucrose solution provided *ad libitum*. When compared to other diet-induced obesity models that have employed high-fat diets without added sugar on the same mouse strain, the present HFHSD model provides similar weight gain and glucose intolerance, but milder fasting hyperglycemia and hyperinsulinemia [15,17]. Altogether, this indicates a less severe metabolic syndrome in mice fed a HFHSD as compared to mice fed the more traditional HFD. Accordingly, memory impairment in the Y-maze test was previously observed in mice under HFD [15] but not in the present study. On the other hand, object recognition tasks still depicted impaired memory performance in the HFHSD-fed mice. By analyzing free open-field exploration, we further found HFHSD-induced impairments in novel environment exploration and increased anxiety-like behavior [25], as was also reported for mice exposed to HFD [28], and in line with a proposed obesity associated depression [29].

In contrast to most diet-induced obesity studies that have focused on male rodents, our study involved mice of both genders. Despite similar weight gain, female mice fed a HFHSD developed a metabolic phenotype that was less severe than in males. However, HFHSD feeding had a similar effect on behavior, that is, both males and females developed memory impairment and anxiety-like behavior. Interestingly, development of hyperinsulinemia was strongly sex-dependent, suggesting insulin levels to be a minor contributor to HFHSD-elicited brain dysfunction. However, the interaction between metabolic syndrome and the gender dimorphism of brain insulin sensitivity remains to be determined [30].

In our study, neuronal loss was not observed in mice fed HFHSD. Indeed, memory impairment in diabetes models is thought to be caused by synaptic dysfunction rather than neuronal loss in the hippocampus [5,6]. Moreover, obesity-independent impairments in hippocampal neurogenesis have been observed in T2D models and proposed to contribute to poor performance in behavioral tasks [31,32]. Adult neurogenesis occurs mainly in the subventricular zone of the lateral ventricles and the subgranular zone of the dentate gyrus within the hippocampus, and has been linked to learning and memory, as well as behaviors related to stress, anxiety and depression [33]. Gender-specific modifications of hippocampal neurogenesis have been observed in mice exposed to HFD from 2 to 6 months of age [34]. The authors propose that deficits in cell proliferation within the dorsal but not ventral hippocampus, which was observed in females but not in males, could contribute to memory decline in females. In contrast, HFD feeding for 4 weeks led to impaired hippocampal neurogenesis in male but not female rats [35]. However, none of the studies reported hippocampal-dependent behavior alterations. Although we did not measure neurogenesis, we analyzed the number of doublecortin-positive cells in the dentate gyrus as immature neurons. The number of immature neurons in the dentate gyrus was not significantly modified, but tended to be reduced in HFHSD-fed mice versus controls. Since factors other than neurogenesis contribute to adequate learning and memory performance, it is likely that cerebral homeostatic alterations triggered by HFHSD feeding lead to similar behavior impairments in male and female mice. Notably, brain dysfunction triggered by HFHSD feeding of mice of either gender was nearly fully recovered upon diet normalization. Recovery of memory impairments in male rats fed a HFD for 3 months after weaning were also partly recovered by diet normalization [36]. Together with the absence of diabetes-induced neuronal death [6], this suggests that the above proposed synaptic dysfunction (not measured in the present study) and reduced neurogenesis in obesity-associated metabolic syndrome are likely to be recovered upon implementation of a healthy diet.

### Alterations in brain metabolism

Mice exposed to a HFHSD showed alterations in the metabolite profile of both hippocampus and cortex. Furthermore, we now established that dimensionality reduction tools (PCA analysis) allowed the prediction of brain metabolite shifts, suggesting that the observed alterations in brain metabolism caused by HFHSD feeding are robust.

The hippocampus appeared to be more sensitive to HFHSD than the cortex, as also reported for HFD feeding [15]. Reduced levels of lactate in the hippocampus of HFHSD fed mice from 4 weeks onward suggest alterations in central energy metabolism. While the reduction in hippocampal lactate levels was sustained until the end of the study, it was not observed in the cortex. Instead, alanine concentration in the cortex was increased after 4 weeks of HFHSD feeding in the absence of lactate changes. We have previously observed a reduction in the phosphocreatine-to-creatine ratio in mice after 6 months of feeding with a fat-enriched diet, when compared to mice fed a diet low in fat [15]. These alterations were not observed in the present study, which is in line with the stronger impact of a HFD on brain energy metabolism, as compared with a diet that also include a high sugar content.

Total creatine content in the hippocampus was increased in mice fed HFHSD, relative to low-fat diet-fed controls, as well as other highly concentrated metabolites, namely taurine, *myo*-inositol and *N*-acetylaspartate, and all recovered upon diet reversal. These metabolites were also found to increase in the hippocampus of insulin resistant rats [11] and HFD-fed mice [15], relative to the respective controls. One might suspect that HFHSD is thus linked to some degree of osmolarity dysregulation [37], which remains to be investigated.

Brain taurine acts as an agonist of receptors involved in GABAergic and glycinergic neurotransmission [38] and plays and important role in preserving mitochondrial function and preventing oxidative damage [39]. In addition to acting as organic osmolyte, increased taurine in the hippocampus of HFHSD-fed mice might thus be a beneficial adaptation, and act as compensatory mechanism for the loss of inhibitory tone and against mitochondrial stress. This beneficial adaptation was not observed in the cortex.

HFHSD-fed mice showed glutathione levels increased in the hippocampus but decreased in the cortex, relative to controls. Similar observations were reported in HFD-fed mice [15]. A previous study found decreased ratio of reduced-to-oxidized glutathione as a consequence of short-term HFD feeding [40]. In contrast, brain levels of both oxidized and reduced glutathione, as well as their ratio, have been shown to be unaffected by long-term HFD feeding [41]. In our study, it is likely that a total glutathione increase in the hippocampus might reflect a compensatory mechanism to oxidative stress upon chronic HFHSD feeding. Interestingly, impaired cellular redox regulation in the brain has been proposed to compromise oligodendrocyte proliferation (consistent with increased levels of *N*-acetylaspartate, discussed below), which has implications on the maintenance of myelin sheets around axons, and thus brain connectivity (see [42], and references there in).

*N*-acetylaspartate is synthesized from neuronal mitochondrial acetyl-CoA and aspartate [43], and its de-acetylation occurs in oligodendrocytes [44]. Therefore, reduced levels of *N*-acetylaspartate have been associated to neuronal injury, and its accumulation might represent oligodendrocyte dysfunction and impaired myelination, such as in Canavans disease (discussed in [37]). In the cortex, *N*-acetylaspartate levels were reduced by HFHSD feeding, indicating neuronal dysfunction. In the hippocampus, *N*-acetylaspartate accumulation could result from reduced aspartoacylase activity in oligodendrocytes, since high-fat feeding was suggested to impact myelination by promoting the loss of oligodendrocyte progenitor cells and mature oligodendrocytes [45].

We also found a HFHSD-induced consecutive increase of hippocampal concentrations of the main neurotransmitter’s glutamate and GABA. This suggests a differential impact of HFHSD feeding on glutamatergic and GABAergic systems. A tendency of increased levels of glutamate, but not GABA, has been reported in previous studies in which mice were fed a HFD for either 2 or 6 months [15,46]. Liu *et al*. [43] reported a HFD-induced increase in the rate of GABA synthesis from glucose without GABA accumulation in the brain. An earlier study on Zucker diabetic rats by Sickmann *et al*. suggested that diabetes has a differential impact on metabolic compartments that produce glutamate and GABA in the hippocampus, but not in the cortex or cerebellum [47]. While T2D has been proposed to affect mainly glutamatergic synapses in NONcNZO/LtJ mice [21], the presynaptic vesicular carriers vGLUT1 and vGAT that are necessary for the function of both neurotransmission systems were affected by long-term HFD feeding [15]. A direct link between glutamate and GABA levels and neurotransmission remains to be determined.

The content of total choline was higher in hippocampus, but not cortex, of HFHSD-fed mice than controls, as has also been observed in HFD-fed mice [15]. The main contributors to the choline peaks in MRS are the water-soluble glycerophosphorylcholine and phosphoryl-choline, which are involved in membrane lipid metabolism, myelination processes and immune responses [37]. Oxidized choline-containing phospholipids produced by oxidative stress have been proposed to contribute to neurodegeneration [48]. Recently, it was also proposed that oxidized choline containing lipids under normal conditions are cleared by microglia, to prevent neuronal injury [49]. Susceptibility to neuronal dysfunction might occur in HFHSD exposure, but taurine and GSH in the hippocampus might prevent oxidation of choline-containing lipids.

Neuroinflammation has also been proposed as an important early step in HFD-induced brain dysfunction [6], namely in the hypothalamus [50-52]. We observed some degree of microgliosis in both the hippocampus and cortex of HFHSD-fed mice but not a striking pro-inflammatory phenotype, which is in line with the small or negligible choline concentration changes.

## Limitations

Others have reported behavior alterations after only one week of HFD exposure [53]. Against our expectations, we did not detect brain dysfunction by analyzing working spatial memory in the Y-maze, which was the behavior test of choice for longitudinal assessments after a pilot experiment using a HFD alone (data not shown). Therefore, the present study does not allow us to directly determine whether metabolic alterations precede neurocognitive dysfunction. We have observed the first sustained metabolic alteration at 4 weeks of HFHSD exposure (reduced lactate in hippocampus), but the Y-maze test revealed no signs of memory impairment at any time point.

Another limitation of the present study is that MRS metabolomics *in vivo* does not determine fluxes. Hence, it is unclear whether alterations in metabolite levels reflect metabolic flux modifications. However, this study still pinpoints metabolic pathways that are affected by HFHSD, which then can be more carefully investigated in future studies.

## Conclusion

Our study revealed robust and systematic alterations in the cortical and hippocampal metabolite profile in HFHSD-fed mice. Metabolite profile shifts indicate *e.g.*, alterations in energy metabolism, adaptations to counteract osmolarity imbalances and mitochondrial stress, and cortical neurodegeneration. Importantly, even though these changes occurred within weeks after initiation of obesogenic diet feeding, we demonstrate an almost complete reversal of the observed phenotype after reinstatement of a healthier diet. Hence, HFHSD-elicited alterations in brain physiology show plasticity, which suggests adaptation rather than permanent structural damage of brain tissue in response to an unhealthy diet.

## Supplementary Materials

The Supplementary data can be found online at: www.aginganddisease.org/EN/10.14336/AD.2021.0720.


