# Supplementary material for: Cognitive Impairment and Metabolite Profile Alterations in the Hippocampus and Cortex of Male and Female Mice Exposed to a Fat and Sugar-Rich Diet are Normalized by Diet Reversal
Source: Aging Dis. 2022 Feb 1;13(1):267–83. doi: 10.14336/AD.2021.0720 (PMC8782561; doi:10.14336/AD.2021.0720)
Supplement: Supplementary file 1 [file AD-13-1-267-s.pdf]

SUPPLEMENTARY DATA

Cognitive Impairment and Metabolite Profile Alterations in the Hippocampus and Cortex of Male and Female Mice Exposed to a Fat and Sugar-Rich Diet are Normalized by Diet Reversal

Alba M. Garcia-Serrano[1,2], Adélaïde A. Mohr[3], Juliette Philippe[1,2], Cecilia Skoug[1,2], Peter Spégel[4], João M.N. Duarte[1,2*]

© 2021. Garcia-Serrano AM. et al. Published online at http://www.aginganddisease.org/EN/10.14336/AD.2021.0720

SUPPLEMENTARY DATA

Supplementary table 1. ANOVA P-values for metabolite concentrations in the hippocampus and cortex during 1 to 16 weeks of HFHSD exposure.

**Metabolite**	**Brain area**	**Interaction**	**Time**	**Diet**
Alanine	hippocampus	**P=0.008**	**P<0.001**	P=0.371
	**cortex**	**P<0.001**	**P<0.001**	**P<0.001**
Aspartate	hippocampus	P=0.501	P=0.764	P=0.256
	cortex	P=0.394	P=0.703	P=0.572
Creatine	hippocampus	P=0.061	**P<0.001**	**P<0.001**
	cortex	P=0.747	**P=0.029**	P=0.431
Phosphocreatine	hippocampus	P<0.001	**P=0.016**	P=0.005
	cortex	P=0.743	**P<0.001**	P=0.857
GABA	hippocampus	**P=0.003**	P=0.200	P=0.199
	cortex	P=0.528	**P=0.047**	P=0.880
Glutamine	hippocampus	P=0.390	**P=0.023**	**P=0.018**
	cortex	P=0.313	**P=0.002**	**P<0.001**
Glutamate	hippocampus	**P<0.001**	**P=0.029**	**P<0.001**
	cortex	P=0.072	**P<0.001**	P=0.421
Glutathione	hippocampus	**P=0.016**	P=0.398	P=0.296
	cortex	P=0.184	**P<0.001**	**P=0.015**
Glycine	hippocampus	P=0.243	P=0.481	P=0.624
	cortex	P=0.542	**P<0.001**	**P=0.002**
myo-inositol	hippocampus	**P=0.026**	P=0.063	P=0.242
	cortex	P=0.750	**P<0.001**	P=0.528
Lactate	hippocampus	**P<0.001**	**P<0.001**	**P<0.001**
	cortex	P=0.367	**P=0.002**	P=0.329
NAA	hippocampus	**P<0.001**	**P<0.001**	**P<0.001**
	cortex	**P=0.033**	**P=0.034**	P=0.113
Taurine	hippocampus	**P<0.001**	**P<0.001**	**P<0.001**
	cortex	P=0.989	**P<0.001**	P=0.281
Ascorbate	hippocampus	P=0.084	**P=0.001**	P=0.888
	cortex	P=0.989	**P<0.001**	P=0.281
Glucose	hippocampus	P=0.484	**P<0.001**	P=0.069
	cortex	P=0.680	**P=0.001**	**P=0.011**
NAAG	hippocampus	**P=0.042**	P=0.364	P=0.151
	cortex	P=0.424	P=0.573	P=0.351
PE	hippocampus	P=0.716	P=0.138	P=0.229
	cortex	P=0.602	**P=0.042**	P=0.474
Total choline	hippocampus	**P=0.042**	**P=0.042**	**P=0.042**
	cortex	P=0.165	**P=0.042**	P=0.632
Total creatine	hippocampus	**P=0.042**	**P=0.042**	**P=0.042**
	cortex	P=0.661	**P=0.042**	P=0.768
PCr/Cr	hippocampus	**P=0.042**	P=0.274	P=0.932
	cortex	P=0.836	**P=0.042**	P=0.560

© 2021. Garcia-Serrano AM. et al. Published online at http://www.aginganddisease.org/EN/10.14336/AD.2021.0720

SUPPLEMENTARY DATA

Supplementary table 2. ANOVA P-values for metabolite concentrations in the hippocampus and cortex at 24 weeks of the study.

Metabolite	Brain area	ANOVA at 24 weeks
Alanine	hippocampus	P=0.966
	cortex	**P=0.042**
Aspartate	hippocampus	P=0.343
	cortex	P=0.580
Creatine (Cr)	hippocampus	P=0.776
	cortex	**P=0.042**
Phosphocreatine (PCr)	hippocampus	**P=0.042**
	cortex	P=0.903
GABA	hippocampus	**P=0.042**
	cortex	P=0.781
Glutamine	hippocampus	P=0.150
	cortex	**P=0.042**
Glutamate	hippocampus	P<0.001
	cortex	P=0.069
Glutathione	hippocampus	**P=0.042**
	cortex	**P=0.042**
Glycine	hippocampus	P=0.205
	cortex	**P=0.042**
myo-inositol	hippocampus	**P=0.042**
	cortex	P=0.138
Lactate	hippocampus	**P=0.042**
	cortex	P=0.058
NAA	hippocampus	P=0.064
	cortex	**P=0.042**
Taurine	hippocampus	**P=0.042**
	cortex	P=0.542
Ascorbate	hippocampus	P=0.433
	cortex	P=0.533
Glucose	hippocampus	P=0.490
	cortex	P=0.644
NAAG	hippocampus	P=0.819
	cortex	P=0.061
PE	hippocampus	**P=0.042**
	cortex	P=0.938
Total choline	hippocampus	P=0.063
	cortex	P=0.326
Total creatine	hippocampus	**P=0.042**
	cortex	P=0.154
PCr/Cr	hippocampus	P=0.122
	cortex	**P=0.042**

© 2021. Garcia-Serrano AM. et al. Published online at http://www.aginganddisease.org/EN/10.14336/AD.2021.0720
